# Postpartum urinary retention: what are the sequelae? A long-term study and review of the literature

**DOI:** 10.1007/s00192-021-05074-5

**Published:** 2022-02-07

**Authors:** Stefan Mohr, Luigi Raio, Ursula Gobrecht-Keller, Sara Imboden, Michael D. Mueller, Annette Kuhn

**Affiliations:** 1 Department of Obstetrics and Gynecology, Inselspital, Bern University Hospital, University of Bern, Friedbühlstrasse 19, 3010 Bern, Switzerland; 2 Department of Obstetrics and Gynecology, Basel University Hospital, Basel, Switzerland

**Keywords:** Postpartum urinary retention, Residual urine, Post-void residual volume, Overt urinary retention, Covert urinary retention, Voiding problems

## Abstract

**Introduction and hypothesis:**

Postpartum urinary retention (PUR) may cause long-term urogenital tract morbidity. The incidence ranges from 0.18 to 14.6%, but the importance of prompt diagnosis and appropriate management is often underappreciated. The paucity of data on long-term outcome after PUR contributes to these drawbacks. The aim of this study was to assess long-term persistence of elevated PVR (post-void residual urine) volume after PUR. Pathophysiology, risk factors and management of PUR are reviewed.

**Methods:**

In our tertiary referral urogynecology unit in the University Women’s Hospital of Bern, Switzerland, all patients who were referred for PUR were asked to participate in this study. PVR was measured sonographically every 2 days until day 15, then after 6, 12, 24 and 36 months and, if increased, the patients were instructed to perform clean intermittent self-catheterization. If retention persisted longer than the lactation period, multichannel urodynamics was performed.

**Results:**

Sixty-two patients were included. The median PVR normalized at day 7. Long-term voiding disorders were found in 8.2%, 6.7%, and 4.9% after 1, 2, and 3 years respectively. Multichannel urodynamics confirmed in all patients with persisting retention an acontractile detrusor and de novo stress urinary incontinence in 4 cases. Quantile regression did not reveal any factor contributing to earlier recovery. Eighty-nine percent of the patients with PUR had operative vaginal deliveries, emphasizing the importance of this risk factor for PUR.

**Conclusions:**

In most cases PUR resolves early, but voiding difficulties persist more often than previously thought, and for these patients the consequences are devastating. Obstetric awareness, early active management, and developing management strategies in the postpartum period might preclude lower urinary tract morbidity.

## Introduction

Postpartum urinary retention (PUR) is a serious and frequent complication after childbirth [1–3]. Overt PUR has been defined as the inability to void within 6 h of delivery or after removal of the catheter, whereas covert PUR means an increased post-void residual urine volume (PVR) of more than 150 ml after spontaneous micturition [1, 4]. The incidence of PUR ranges from 0.18 to 47% [1–16] depending on the varying definitions and the time interval of follow-up used.

The pathophysiology of PUR is poorly understood. Several theories have been suggested, including physiological, neurological, and mechanical processes during pregnancy and delivery [1, 2, 6]. Levator ani muscle avulsion seems to be associated with persistent postpartum voiding dysfunction [17], but the etiology of PUR is thought to be multifactorial. Trauma to the pelvic floor muscles, the detrusor muscle itself, and overdistention of nerve fibers might impair bladder sensitivity, cause periurethral obstructing edema, and hormonal changes may influence bladder function as well [1, 2]. PUR can lead to denervation, detrusor atony, and bladder dysfunction if it is not recognized in time [2, 13, 16, 18]. Although long-term consequences of PUR are rarely reported [19] and small studies showed negligible if any clinical impact on long-term urogynecological disorders [19], only a few data are available on the potential long-term micturition problems of increased PVR after vaginal delivery [20]. Persistent urinary retention can be a serious condition for the patient and requires management in order to prevent urogenital tract morbidity such as micturition problems due to detrusor failure, kidney failure, anuria, and hydronephrosis [1, 2], even in later life [16]. Complications such as urothelial lesions [21] and urinary bladder rupture have been described [22, 23]. Prompt diagnosis and appropriate management are the key to restoring normal bladder function [1, 2], but recognition is hampered by a low level of awareness amongst obstetric units and a scarcity of published literature [24].

Risk factors for PUR include: a prolonged second stage of labor [2, 4–6, 8, 18, 25–29] (duration of labor [first and second stage] >700 min predicts PUR [8, 30]), vacuum-assisted [2, 25], and instrumental delivery [5, 6, 8–11, 14, 23, 26, 27, 29, 31, 32], (high grade) perineal lacerations [3, 6, 10, 13, 18, 25–27, 29, 31, 33], and episiotomy [5, 9, 11, 16, 18, 23, 33], degree of perineal pain [5], fetal birth weight [6, 16, 18, 32], use of systemic narcotics [16, 29], nulliparity [3, 5, 6, 9, 11, 13, 23, 25–27], cesarean section (possibly after failure to progress in labor) [12, 13, 28, 32, 33], epidural analgesia (possibly by modifying other obstetrical parameters such as duration of labor) [5, 7, 9, 15, 16, 23, 25–27, 29, 32], intermittent catheterization during labor [3], an increasing number of catheterizations [34], and an absence of spontaneous voiding before leaving the delivery room [31]. One study identified urination just before delivery as a preventive factor for PUR [11].

Commonly, PUR is thought to be transient [1, 4], possibly because PVR is not routinely measured and therefore PUR is underdiagnosed, but data to sustain the transient nature of PUR are scarce [1, 20].

In particular, there is a paucity of data on long-term outcome after postpartum urinary retention and time period to normalization of post-void residual urine volume. The aim of this study was to assess long-term persistence of elevated post-void residual urine volume after PUR. We hypothesize that postpartum urinary retention does not necessarily resolve spontaneously in all women.

## Materials and methods

We performed a prospective cohort study in our tertiary referral urogynecology unit in the University Women’s Hospital of Bern, Switzerland. In our Department 2,300 babies are delivered annually and we have 65 gynecological beds. The rate of vaginal operative delivery is 13% in our hospital. Patients who were referred to our unit for immediate PUR from our own maternity ward, as well as from outside hospitals, were asked to participate in this study. All patients were referred on the day of or after delivery because the PVR was greater than 500 cc or the patient was symptomatic or not able to void at all. Ethical consent from the local ethics committee was obtained (Kantonale Ethikkommission Bern, 16-04-2008) and patients were asked to sign consent forms. The primary outcome was the time to normalization of post-void residual urine, the secondary outcomes were influencing factors for persistent increased volumes.

At first visit, demographic data, obstetric data such as mode of delivery, birth weight, type of anesthetic, and obstetric complications were noted.

Post-void residual urine was measured using ultrasound (Aloka Systems, Japan) applying the curved array 3.5-MHz probe immediately after micturition with the formula width × length × depth × 0.6 [35]. Residual urine was defined as significant if more than 150 ml were measured. Immediately after the diagnosis of retention was made a permanent catheter was placed for 24 h, then removed, and patients were instructed to perform clean intermittent self-catheterization afterwards.

Self-catheterization was instructed by specialized incontinence nurses in an outpatient setting. Catheter choice and the patient’s position during catheterization were performed according to patients’ preference and manual abilities, and catheter intervals were prescribed according to the amount of residual urine, aiming at not surpassing the bladder capacity of more than 500 ml. Patients were catheterized depending on residual urine volume, i.e., once a day over 100 ml, twice a day over 200 ml, and so forth. At follow-up, difficulties using the self-catheterization were noted. Patients were asked to fill in a voiding-residual diary. In case of an inability to self-catheterize, a transurethral Foley catheter was offered.

Initially, residual was measured every 2 days until day 15, then after 6 months and 12 months. After this, patients were asked to attend our clinic for follow-up on a yearly basis.

In cases of retention that lasted longer than the lactation period, multichannel urodynamics was performed according to ICS recommendations [36]. Briefly, multichannel urodynamics were performed in the sitting 45º upright position. Filling was continued until the patient experienced a strong desire to void. At bladder capacity pressure flow studies were performed. Side effects of self-catheterization, de novo symptoms, and urinary tract infections were noted. For statistical analysis, GraphPad Prism, version 6.0 for Windows, was used. Additionally, both Cox regression and quantile regression were used to explore factors influencing time to normal urinary retention (≤150 ml). Quantile regression estimates how much a predictor influences the median of the variable of interest, in this case, time to normal urinary retention. These analyses were performed with Stata 16.1, StataCorp 2019.

## Results

All 62 patients who were asked to participate were included in this study. Median age was 29 years (range 17 to 45). Median body mass index (BMI) was 27 kg/m^2^ (range 19 to 34). Parity was 1 in 37 women, 2 in 12 patients, 3 in 5 women, parity 5 in 4 women and 8 in 3 women. For 1 patient demographic data were not retrievable.

Delivery mode was instrumental in 88%: forceps extraction in 8, vacuum-assisted delivery in 43, and spontaneous delivery in 6 patients, and 1 had a cesarean section. In 4 patients the delivery mode was not retrievable. Sixty-one percent had an epidural anesthesia and 10 % had a pudendal block.

Gestational age at delivery was 39 + 2 weeks (range 33 + 0 to 41 + 2). Median blood loss during delivery was 500 ml (range 200–1,500 ml). Mean duration of the first stage of labor was 432 min (range 189–670) and of the second stage it was 190 min (range 20–305) respectively. Birthweights showed a median of 3,900 g (2,900–4,680). Head circumference was a median of 36 cm (range 31–39). All women had singleton deliveries.

**Fig. 1:**
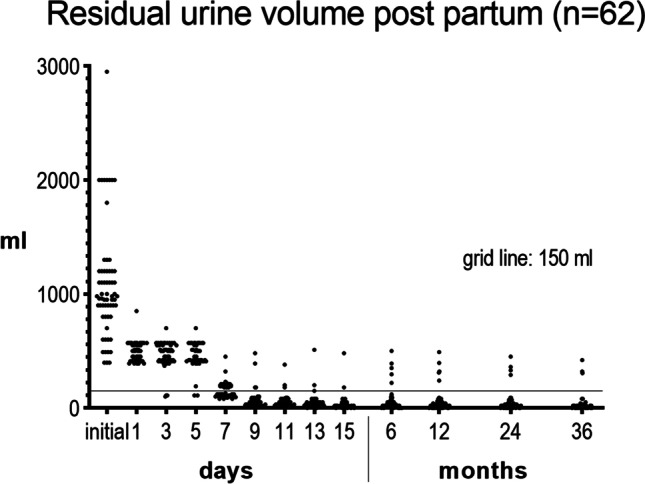
Development of residual urine volume postpartum, i.e., measured every 2 days until day 15, then after 6, 12, 24 and 36 months

The initial PVR was ≥2,000 ml in 8 patients of whom 5, 4, and 3 had increased PVR after 12, 24, and 36 months respectively. The median residual volume postpartum normalized (i.e., went below 150 ml) at day 7 (Fig. 1,Table 1). Long-term voiding disorders (i.e., PVR > 150 ml) were found in 5 (8.2%), 4 (6.7%), and 3 (4.9%) of patients after 1, 2, and 3 years respectively. Multichannel urodynamics confirmed in all patients with persisting retention an acontractile detrusor and de novo stress urinary incontinence in 4 cases. Lost-to-follow-up measurements were one woman after 12, two women after 24, and one woman after 36 months respectively, and these women were excluded from the respective analyses at these time points.

**Table 1:** Median residual urine volume measures in milliliters

	Initial	Day								Month			
		1	3	5	7	9	11	13	15	6	12	24	36
Minimum (ml)	399	390	100	110	80	20	20	0	0	0	0	0	0
Median (ml)	980	500	500	435	**120**	**50**	50	30	20	22.5	30	30	20
Maximum (ml)	2,950	850	700	700	450	480	380	510	480	500	490	450	420
Number of patients with PVR ≥ 150	62	62	59	58	37	5	3	2	2	5	5	4	3

(Bold: median volumes decreasing below 150ml and 100ml, respectively, on day 7)

Quantile regression results for age, parity, BMI, spontaneous delivery, length of first and second stages of labor, birth weight, head circumference, and epidural were of no value owing to little variation in the data, partly due to the sampling method of sampling every 2nd day. However, the only factor found to be of statistical significance was spontaneous birth (median −2.00, 95% CI −2.22 to −1.78, *p* < 0.001). Spontaneous delivery seems to reduce the time to normal PVR by a median of 2 days (Tables 3).

## Discussion

Our study shows that although being transient, in most cases persistent voiding disorders after postpartum urinary retention are not negligible, and if increased PVR does not resolve by day 7 postpartum it is likely to persist. Our findings show that 8.2%, 6.7%, and 4.9% of the women have long-term voiding difficulties after 1, 2, and 3 years respectively, requiring intermittent clean self-catheterization after postpartum urinary retention (Fig. 1, Table 1). In the majority of cases normal voiding was re-established after 7 days, and patients with long-term voiding disorder had noticeably high initial residual volumes of >2,000 ml that reportedly persisted over several hours and were managed poorly with in-and-out catheters instead of adequate continuous drainage. Yet, for high initial residual volumes being a risk factor we were not able to demonstrate statistical significance, possibly because of the lack of variance in our data and the rare event of persistent long-term voiding problems.

Post-void residual volume is not uniformly defined and there is no consensus on what constitutes a significant elevation or at which volume PVR contributes to urinary problems [37–39]. The current cut-off values for covert PUR are rather arbitrary until data on the clinical consequences of abnormal PVR are available [16]. The arbitrary cut-off of 150 ml in nonpregnant women does not exclude voiding dysfunction [40]. The Agency Health Care Research and Quality guidelines define a PVR of less than 50 ml as being indicative of adequate emptying and a PVR of more than 200 ml as inadequate emptying [37], but the range between 50 and 200 ml remains ambiguous. Stricter definitions use a PVR cut-off of 100 ml or one third of total volume, in contrast to 150–200 ml or one half of total volume [41]. In men, covert urinary retention definitions range from 300 to 1,000 ml, reflecting the lack of clear cut-offs [42]. A change of the current definition of covert PUR was proposed to be ≥500 ml after the first postpartum void because above this cut-off some women do need more time to normalize emptying of the bladder [20].

Previous research has shown that only two thirds of women achieve normal voiding within 4–14 days and another fifth within 15–28 days postpartum [2]. In most cases PUR resolves early (before hospital discharge [9] or within 4 days [4, 20]), but PUR also can persist for a median of 19 (range 3–85 [7] or 1–45 [1]) days. Higher PVR 3 days after delivery is associated with increased risk for late recovery [2], which argues for early diagnosis and timely intervention avoiding long-term consequences [2]. Although covert retention was formerly stated to be self-limiting and specific treatment considered unnecessary [4], rates of protracted, persistent, or chronic PUR of 0.11% [9], 0.18% [2], 0.18% [10], and 0.2% [7] are nevertheless alarming.

In our patients, the rate of persistent PUR was remarkably higher at 8.2% after 12 months, 6.7% at 24 months, and 4.9% after 3 years (Fig. 1, Table 1). One explanation may be the high number of patients with very large initial residual volumes of more than 1,000 ml (*n* = 29). These results are in contrast to those of Mulder et al., who stated that persistent PUR is common and transient and does not result in more lower urinary tract symptoms 1 year after delivery [20]. Although the absolute number of patients suffering from protracted PVR in the long term is low, the consequences for these individuals are immense.

Data on the treatment of postpartum urinary retention are scarce. Cholinergic medication was already suggested in 1957, but is known to have insufficient efficacy and results in side effects today [43]. PUR is commonly treated with (repeated) catheterization and/or instruction in intermittent self-catheterization [1, 7, 10]. Clean intermittent self-catheterization is preferred to indwelling catheterization, well tolerated, and not inferior regarding development of micturition symptoms [44–46]. Administration of antibiotics during catheterization is not beneficial and is not recommended [47].

In contrast to postoperative management in gynecological surgery, routine measurement of the PVR is not established in obstetrics [1, 23], and the lack of guidelines is one of the major problems in treating women with PUR [1, 48, 49]. Systematic sonographic monitoring of all postpartum patients at least until day 3 [15, 49] and for 24 h after epidural analgesia [7] has been recommended to avoid excessive urinary retention. The most precise technique for measuring PVR is bladder catheterization [50], but, owing to being less invasive, both portable bladder scanning devices and 2D/3D ultrasound are to be favored [15, 51, 52], particularly because ultrasonic assessment is accurate enough to be used for making clinical decisions [53–55] and to screen women for residual volume in particular [51]. However, conflicting results exist regarding the accuracy of specific devices [50, 52].

As routine PVR measurements are time consuming and costly, a risk-factor-based approach has been proposed to only include patients at risk in purposeful sonographic monitoring [18], although this approach is controversial [13]. Knowledge of the risk factors might help to develop preventive measures [18, 31], improve early identification of women at risk, and lead to enhanced postpartum surveillance [3, 25], but further studies are needed to assess the efficiency of early systematic bladder scanning in patients with risk factors for PUR [31].

In line with the risk factors outlined in our introduction, the high rate of vaginal operative deliveries of 88% in our patients leads to the assumption that forceps and vacuum extraction are associated with PUR. Time until diagnosis/management and urinary volume at first catheterization after delivery have been pointed out as risk factors for persistent PUR in particular [33], which again argues for attentive monitoring of postpartum voiding to minimize long-term complications. The analysis of our patients did not find any correlation between normalization of PVR after day 7 and 9 respectively, regarding age, BMI, spontaneous delivery, duration of 1st and 2nd stages of labor, birthweight, head circumference, duration of pregnancy, and epidural use (Table 2). Of note, these factors were analyzed regarding their influence on the time period to normalization of PVR and not as risk factors for PVR in general. Regarding the risk for prolonged PUR, we did not find increased hazard ratios for the above-mentioned factors (Table 3), but quantile regression revealed that in women with spontaneous delivery, the time to PVR normalization was reduced by 2 days compared with instrumental delivery. However, owing to the large number of ties (multiple events occurring at the same time point), Cox regression is unlikely to be very accurate.

**Table 2:** Demographic and peripartum factors (left column) grouped by normalization of post-void residual urine at 7 and 9 days. *p* values were calculated using Fisher’s exact test and Wilcoxon/Mann–Whitney tests respectively, to compare if there were differences in the grouped factors between patients with normalized PVR and those with persistent PVR (i.e., if there were differences in age distribution between patients with normalized and those with persistent PVR)

	Total (*n* = 62)	PVR > 150 ml by day 7 (*n* = 44)	PVR ≤ 150 ml by day 7 (*n* = 18)	*p* value day 7	PVR > 150 ml by day 9 (*n* = 4)	PVR ≤ 150 ml by day 9 (*n* = 58)	*p* value day 9
	Median (lq, uq) or *n* (%)	Median (lq, uq) or *n* (%)	Median (lq, uq) or *n* (%)		Median (lq, uq) or *n* (%)	Median (lq, uq) or *n* (%)	
Age (years)	29 (23, 35)	29 (23, 35)	34 (23, 37)	0.63	37 [27, 41]	29 [23, 35]	0.23
BMI (kg/m^2^)	27 (21, 29)	27 (21, 29)	28 (27, 29)	0.33	29 (29, 34)	27 (21, 29)	0.030
Spontaneous delivery				0.14			0.12
No	52 (84%)	39 (89%)	13 (72%)		2 (50%)	50 (86%)	
Yes	10 (16%)	5 (11%)	5 (28%)		2 (50%)	8 (14%)	
1st stage of labor (min)	390 (320, 580)	343 (320, 580)	470 (340, 580)	0.64	508 (343, 670)	390 (320, 580)	0.19
2nd stage of labor (min)	190 (90, 200)	170 (90, 198)	190 (90, 210)	0.83	195 (193, 198)	150 (90, 210)	0.22
Birthweight (kg)	3.9 (3.5, 4.1)	3.9 (3.4, 4.1)	3.7 (3.5, 4.0)	0.67	3.9 (3.8, 4.2)	3.8 (3.3, 4.1)	0.33
Head circumference (cm)	36 (35, 36)	36 (35, 36)	36 (35, 36)	0.97	36 (36, 37)	36 (35, 36)	0.40
Duration of pregnancy (days)	275 (263, 280)	267 (258, 280)	278 (263, 280)	0.45	273 (259, 282)	275 (263, 280)	0.93
Epidural				0.27			1.00
No	24 (39%)	19 (43%)	5 (28%)		1 (25%)	23 (40%)	
Yes	37 (60%)	24 (55%)	13 (72%)		2 (50%)	35 (60%)	

*BMI* body mass index, *lq* lower quartile, *uq* upper quartile, *PVR* post-void residual

**Table 3:** Hazard ratios (HR) for demographic and obstetric parameters. No factor significantly influenced time to normalization of residual urine volume

	HR (95% CI)	*p* value
Age (years)	1.00 (0.97–1.03)	0.864
Parity	1.00 (0.85–1.16)	0.969
Parity >1	0.93 (0.56–1.56)	0.780
BMI (kg/m^2^)	1.00 (0.94–1.07)	0.931
Spontaneous delivery	1.04 (0.50–2.16)	0.915
1st stage of labor (per 10 min)	1.00 (0.99–1.02)	0.968
2nd stage of labor (per 10 min)	1.00 (0.96–1.04)	0.830
Head circumference (per 5 cm)	0.93 (0.44–1.94)	0.839
Epidural analgesia	1.11 (0.66–1.86)	0.688

*BMI* body mass index, *CI* confidence interval

The idea of PUR being transient and self-limiting [1] has to be questioned. This belief might explain why postpartum urinary retention management guidelines are not yet established [1] but urgently needed in clinical practice [48]. Data on PUR management are insufficient, particularly because treatment options differ widely between studies. Protocols of timed voiding and routine measurement of PVR have been shown to reduce PUR [10, 13, 56]. Even if routine screening is not yet recommended [1], adequate symptom assessment by the health care provider after delivery is of utmost importance [23, 48, 57]. Last but not least, knowledge of PUR is considered to be low amongst residents in obstetrics and gynecology and management guidelines increase their comfort in dealing with patients in the postpartum period [58].

The strength of the current study is the rather large number of patients who have meticulously been followed up for 36 months; a weakness might be that we did not perform repetitive multichannel urodynamics to indicate when detrusor contractility did resume. However, with urodynamics being an invasive and expensive technique that has little value for therapeutical options in urinary retention we considered additional urodynamics as not really helpful. A further weakness is that the results reflect our single-center experience only.

In summary, long-term follow-up data show that acute PUR may have long-term consequences for the patients’ voiding abilities [1]. It is not known if normalization of the PVR implies that the patient does not develop symptoms or complications in later life [1]. The longer acute retention lasts without treatment, the more likely it is that the detrusor muscle is transformed into noncontractile fibrosis [59], which explains acontractility in our patients and potentially in later life. Misdiagnosis or delay in diagnosis of PUR can cause bladder overdistension, leading to irreversible detrusor damage. This argues for careful monitoring and early diagnosis of PUR to prevent immediate and long-term sequelae [6], particularly amongst women who underwent operative vaginal deliveries according to the high rate of operative vaginal deliveries in our cohort.

Future research needs to compare expectant and active management of PUR before stating that covert PUR does not need treatment [1]. Management strategies, adverse effects of PUR, catheterization methods, whether catheterization prevents morbidity, and the necessity for screening and treating PUR need to be studied [1, 10]. Until then, unifying clinical practice and increasing obstetric awareness of this common condition is indicated [1, 56], because, according to our results, long-term morbidity of PUR is rare but has devastating consequences for the few women suffering from voiding difficulties.
